# Host–Pathogen–Vector Continuum in a Changing Landscape: Potential Transmission Pathways for *Bartonella* in a Small Mammal Community

**DOI:** 10.1002/ece3.71085

**Published:** 2025-04-02

**Authors:** B. R. Ansil, Ashwin Viswanathan, Vivek Ramachandran, H. M. Yeshwanth, Avirup Sanyal, Uma Ramakrishnan

**Affiliations:** ^1^ National Centre for Biological Sciences Tata Institute of Fundamental Research Bangalore Karnataka India; ^2^ Manipal Academy of Higher Education Manipal Karnataka India; ^3^ Nature Conservation Foundation Mysore Karnataka India; ^4^ Wildlife Biology and Conservation Program National Centre for Biological Sciences Bangalore Karnataka India; ^5^ Trivedi School of Biosciences Ashoka University Sonipat Haryana India

**Keywords:** bacterial zoonosis, coevolution, ectoparasites, host shifts, vector‐borne pathogens

## Abstract

Bacterial infections account for a large proportion of zoonoses. Our current understanding of zoonotic spillover, however, is largely based on studies from viral systems. Small mammals such as rodents and their ectoparasites present a unique system for studying several bacterial pathogens and mapping their spillover pathways. Using *Bartonella* spp. (a Gram‐negative bacteria) as a model system within a rainforest human‐use landscape, we investigated (1) ecological correlates of *Bartonella* prevalence in small mammal hosts and (2) evolutionary relationships between *Bartonella* spp. and various hosts and ectoparasites to gain insight into pathogen movement pathways within ecological communities. We detected *Bartonella* in five out of eight small mammal species and in 86 (40.56%) out of 212 individuals, but prevalence varied widely among species (0%–75.8%). Seven of the ten ectoparasite species found on these small mammals were positive for *Bartonella*. Interestingly, while *Bartonella* genotypes (15) in small mammals were host‐specific, ectoparasites had nonspecific associations, suggesting the possibility for vector‐mediated cross‐species transmission. We also found that *Bartonella* prevalence in hosts was positively correlated with their aggregated ectoparasite loads, further emphasizing the crucial role that ectoparasites may play in these transmission pathways. Our cophylogenetic analysis and ancestral trait (host) reconstruction revealed incongruence between small mammal and *Bartonella* phylogenies, indicating historic host shifts and validating the potential for contemporary spillover events. We found that small mammal hosts in this fragmented landscape often move across habitat boundaries, creating a transmission pathway (via shared ectoparasites) to novel hosts, which may include synanthropic species like *Rattus rattus* . Our results highlight the necessity to disentangle the complex relationship among hosts, ectoparasites, and bacterial pathogens to understand the implications of undetected spillover events.

## Graphical abstract

This study focuses on *Bartonella* spp. (a Gram‐negative bacteria) in small mammals and their ectoparasites within a rainforest human‐use landscape. We found that a high proportion (40.56%) of the tested small mammals across five species and two sites carry *Bartonella*, with prevalence being positively correlated with aggregated ectoparasite load. Despite the occurrence of host‐specific genotypes, we observed incongruence in small mammal and *Bartonella* phylogenies, indicating historic host shifts within the community, and the potential for cross‐species transmissions in the future mediated by ectoparasites.

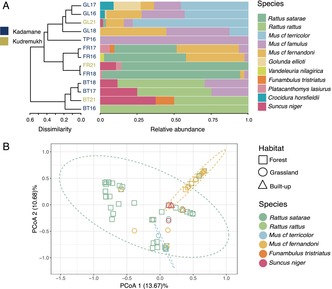

## 1 Introduction

Large‐scale modification of natural habitats is associated with loss of biodiversity and ecosystem services (Dirzo et al. 2014). But these are not the only consequences. Other impacts, such as an elevated risk of zoonotic infections, are often overlooked and remain poorly understood, particularly in biodiverse tropical ecosystems. Both theoretical and empirical studies demonstrate that land‐use change and associated habitat perturbations can result in modified communities of zoonotic hosts driven by the movement of animals across habitat boundaries (Gibb, Redding, et al. 2020; Hassell et al. 2017). Consequently, there is potential for pathogen spillover among animals that did not previously co‐occur, that is, from reservoir hosts to incidental hosts, directly or indirectly through intermediate hosts and vectors (Borremans et al. 2019; Faust et al. 2018).

Pathogen spillover among wildlife can lead to the emergence of zoonotic potential (Borremans et al. 2019) and has been observed in several past epidemics, including SARS‐CoV‐1 (Wang and Eaton 2007). Such processes are often facilitated by the recombination and evolution of new variants with altered host ranges, vector specificities, and pathogenic potential (Pérez‐Losada et al. 2015). Spillover events, however, are complex and infrequent, influenced by numerous ecological and evolutionary filters that vary in space and time, including the distribution of reservoir hosts, pathogen prevalence, and infection intensity (Plowright et al. 2017). A high density of reservoir hosts, for example, elevates direct interactions, leading to density‐dependent transmission among the reservoir population and to other susceptible members of the community (Rodríguez‐Pastor et al. 2019). Additionally, in the case of vector‐borne pathogens, ectoparasites also respond to host densities and influence pathogen prevalence in reservoirs and incidental hosts (Stafford et al. 1998).

Bats and nonvolant small mammals (rodents and shrews—referred to as small mammals hereafter) are the best known reservoir taxa for zoonotic pathogens (Luis et al. 2013). Small mammals harbor a high number of pathogens (Han et al. 2015; Johnson et al. 2020) owing to their disproportionately high diversity within mammalian communities (Burgin et al. 2018), even more so than bats. Small mammals also differ from bats in that they often increase (rather than decrease) in abundance in altered landscapes, resulting in the elevated prevalence of the pathogens they carry (Andreazzi et al. 2023; Morand et al. 2015). Their densities can also increase locally following large herbivore extinctions, further elevating pathogen prevalence and the risk of zoonotic transmission (Allan et al. 2003; Young et al. 2014). In addition, small mammals with generalist life histories (often reservoirs) tend to move across habitat boundaries where they interact with incidental hosts (Borremans et al. 2019; Estavillo et al. 2013), thereby moving pathogens across these boundaries. We expect such processes to be especially prevalent in tropical regions, as they harbor some of the richest small mammal communities (García‐Peña et al. 2021), the highest rates of habitat modification (Brooks et al. 2002; Ellis‐Cockcroft and Cotter 2014), and therefore the highest predicted risk of zoonotic infections (Allen et al. 2017; Jones et al. 2008).

To understand the zoonotic consequences of small mammals and their ectoparasites in changing landscapes, we must first understand how their communities are structured and how they have responded (and continue to respond) to land‐use change and habitat modification. In this study, we characterize differences in host community structure, ectoparasite community structure, and pathogen prevalence in a human‐dominated rainforest plantation landscape in South India, a part of the Western Ghats biodiversity hotspot. We used *Bartonella*, a generalist zoonotic bacterial pathogen, as a model system to understand correlates of pathogen prevalence and potential transmission pathways in the landscape. We sampled small mammal communities and their ectoparasites in this landscape to address the following questions—(i) How are communities of small mammals and their ectoparasites structured? (ii) How does *Bartonella* prevalence vary among small mammal and ectoparasite species? (iii) What are the main correlates of *Bartonella* prevalence in small mammals? (iv) and finally, do we find evidence for spillover or host shifts detected as shared *Bartonella* genotypes among members of the host community?

## 2 Materials and Methods

### 2.1 Field Sampling

**FIGURE 1:**
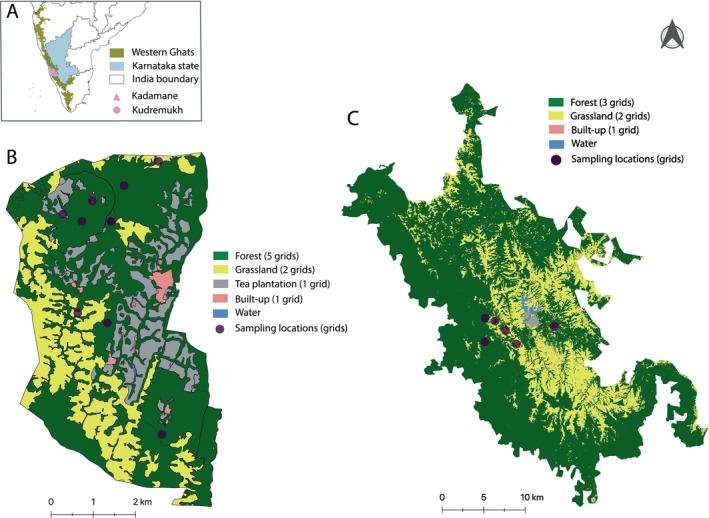
Map of the study area. (A) Map of south India with Karnataka state, Western Ghats and study sites marked. (B) Land‐use map of Kadamane forest‐plantation mosaic with sampling locations marked. (C) Land‐use map of Kudremukh National Park with sampling locations marked.

We carried out this study at similar elevations (900–1100 m asl) in two sites with histories of intense human use in the central Western Ghats of Karnataka state in southern India: (i) Kadamane, a mixed‐use, forest–grassland–plantation mosaic, and (ii) Kudremukh, a forest‐grassland mosaic that is a protected National Park (Figure 1). Both Kadamane and Kudremukh are, however, less disturbed today (Krishnaswamy et al. 2006; Ramachandra et al. 2015). Some plantations in Kadamane have been “rested” over the years and have since rapidly recovered to be near indistinguishable from forest. We therefore considered these rapidly recovering ecosystems also as “forest” in this study. We sampled small mammals (rodents and shrews) from different land‐use types in Kadamane and Kudremukh between 2016 and 2021. In Kadamane (2016, 2017 & 2018), we sampled in forest, grassland, tea plantation, and built‐up habitat (human habitation). In Kudremukh (2021), we sampled in forest, grassland, and built‐up habitat (a partially occupied township now inside the National Park). At both sites, we conducted sampling during the dry season (January–April). We sampled at these two sites not to draw comparisons between them but to get a better representation of small mammals, ectoparasites, and pathogen communities in the region.

We used medium‐sized Sherman traps in a grid framework to sample small mammals. A trapping grid consisted of 10 trapping lines, each line consisting of 10 traps separated by 10 m, covering an area of one hectare in total in each habitat type. An additional 20 traps were placed on trees to capture arboreal species in forests and rested plantations. Trapping efforts were proportionately divided based on the area available under each land‐use category (Figure 1). Specifically, in Kadamane, this translated to five grids in forest, two grids in grassland, one grid in tea plantation, and one grid in built‐up habitat. In Kudremukh, three grids were placed in forest, two in grassland, and one in built‐up habitat.

Each grid was sampled for four consecutive days during each trapping session (assuming a closed population). All traps were baited using peanut butter and locally available seeds. Traps were checked twice a day (morning and evening) for captured individuals. Captured individuals were anesthetized using Isoflurane for collecting measurements and samples. Small mammal species were first identified in the field with the help of taxonomic keys and field guides (Ellerman 1961; Johnsingh and Manjrekar 2015; Menon 2014). In addition, ear clippings were collected to confirm species identification through molecular methods. We collected dried blood spots from every captured individual in 2018 and 2021 to screen for pathogens. To detect and collect ectoparasites, captured individuals were combed on a white paper for 2–3 min. All individuals were marked and released at the site of capture after sample collection (except in 2018 when individuals were sacrificed for harvesting organs to screen for various pathogens).

### 2.2 Small Mammal Community

DNA was extracted from ear clippings using the DNeasy Blood & Tissue Kit (Qiagen), following which 1140 bp of the Cytochrome b (*Cytb*) gene was amplified and sequenced (He et al. 2010). Sequences were aligned using MUSCLE in MEGA 7.0.26 (Kumar et al. 2016). A maximum likelihood phylogenetic tree was constructed using IQ‐TREE 1.6.12 (Nguyen et al. 2015) to identify cryptic *Mus* species and validate morphological species identification. All the other species were identified morphologically and validated using BLAST (blast.ncbi.nlm.nih.gov) similarity scores (97%–100%) of *Cytb* sequences (Meiklejohn et al. 2021). Based on the species identification, we created a species abundance matrix for each land‐use type, followed by a distance matrix using the Morisita–Horn index, a standard beta diversity measure to calculate dissimilarity (Wolda 1981). The beta diversity index was plotted following hierarchical clustering to visualize the dissimilarity among small mammal communities in different land‐use types and sampling years. We also calculated the relative abundance of each small mammal species in each land‐use type to understand species–habitat associations. The community ecology package Vegan in R was used for this analysis (Oksanen et al. 2019; R Core Team 2020).

We used a capture–mark–recapture approach (Seber 1982) to estimate densities of the four most common small mammals by creating individual capture histories in forest and grassland. We assumed a closed population with varying capture probabilities to estimate population size following a customized log‐linear model (Baillargeon and Rivest 2007). Density (number of individuals per hectare) for each species was estimated after accounting for the number of grids sampled in each habitat. We used the R package *Rcapture* for this analysis (Baillargeon and Rivest 2007).

### 2.3 Ectoparasite Community

Ectoparasites were brought back to the laboratory in 100% ethanol. Mites, ticks, and fleas were segregated using a stereo‐zoom microscope. We identified them as morphotypes (potentially species) belonging to specific genera using a high‐resolution microscope with the help of taxonomic keys and monographs (Elango 2022; Geevarghese and Mishra 2011; Iyengar 1973; Mullen and OConnor 2019). Both adult and larval stages of each taxon were considered while identifying morphotypes, thereby reducing the risk of misidentification that might arise from differences in life stage characteristics. We serially named each morphotype under the same genus and counted individual ectoparasites that belonged to the same morphotypes.

We constructed a distance matrix based on ectoparasite occurrence per individual small mammal species (Bray and Curtis 1957). We used this matrix for a Principal Coordinate Analysis (PCoA) in R (R Core Team 2020), and plotted the clustering using ggplot2 (Wickham 2011) to understand the overlap in ectoparasite communities among small mammal species (the first two PCoA axes). The likelihood of ectoparasite occurrence (mites, ticks, and fleas) for each combination of host species, habitat, and site was calculated as the aggregated ectoparasite load. This metric represents the cumulative infestation rate for all ectoparasites. We opted not to use the term “infestation rate” here because it generally refers to the rate specific to each parasite species and can have different interpretations. We also estimated ectoparasite density as the mean number of each morphotype per host species in forest, grassland, and built‐up habitat.

### 2.4 *Bartonella* Screening and Prevalence Estimation

To detect *Bartonella* in small mammals, DNA was extracted using the QIAamp DNA Mini Kit (Qiagen) from the dried blood spots (DBS) collected in 2018 (Kadamane) and 2021 (Kudremukh), and screened for the *Bartonella*‐specific *rpoB* gene (825 bp) using conventional PCR (Renesto et al. 2001). *Bartonella* prevalence in the Kadamane samples was reported previously (Ansil et al. 2021). To detect *Bartonella* in every ectoparasite morphotype found on every small mammal, 1–10 individual ectoparasites (mean = 3.06) from each morphotype were pooled, mechanically homogenized using disposable plastic pestles, and DNA was extracted using the DNeasy Blood & Tissue Kit (Qiagen). We used PCR reagents as follows—25 μL reaction mixture composed of 6 μL of template DNA, 12.5 μL of 1X HotStarTaq Master Mix (Qiagen), 1 μL each of 5 μM primers, and 4.5 μL of nuclease‐free water. PCR products were visualized on a 1.5% agarose gel stained with GelRed Nucleic Acid Gel Stain (Biotium Inc) with reference to negative (PCR reagents without small mammal DNA) and positive controls (*Bartonella* positive sample previously sequenced and confirmed). Positive samples were purified using AMPure XP magnetic beads (Beckman Coulter) and sequenced. Sequences were verified for *Bartonella* after comparing them with the reference database (NCBI) using BLAST (Altschul et al. 1990). Additionally, other regions in the *Bartonella* genome such as *ftsZ* (~900 bp) and *16SrRNA* (369 bp) were also sequenced (Paziewska et al. 2011; Zeaiter et al. 2002) and included in the phylogenetic analysis. All *Bartonella* sequences generated in this study have been deposited in GenBank under accession numbers OR574780 –OR574826, PP800974 –PP800985, PP799247 –PP799254.

We calculated prevalence as the proportion of positive individuals against the number of individual small mammals tested. We calculated 95% confidence intervals associated with the prevalence proportion using the R package *Hmisc* (Harrell Jr 2019). Positivity in ectoparasite pools was represented as the proportion of positive pools among the total number of ectoparasite pools tested.

### 2.5 Ecological Correlates of *Bartonella* Prevalence

We used a binomial generalized linear model (GLM) to understand drivers of *Bartonella* prevalence among small mammals in the landscape. We modeled prevalence in each small mammal species as a function of the sampling site, the density of the species, and aggregated ectoparasite load (the proportion of all individuals of the species with any ectoparasite presence—mites, ticks, and fleas—within the relevant habitat‐site combination). While only a subset of these parasites may be actively involved in transmission, we aggregated them because previous research indicates that several of the relevant ectoparasite genera, including *Laelaps*, *Ixodes*, *Haemaphysalis*, and *Xenopsylla*, are involved in *Bartonella* transmission (Jiyipong et al. 2014; Kaminskienė et al. 2022). Moreover, the covariate ‘aggregated ectoparasite load’ is not the ectoparasite density per individual small mammal, but the frequency of ectoparasite occurrence in each small mammal species, habitat, and site. We considered this a more reliable measure than raw ectoparasite density since it is possible to occasionally encounter individuals with heavy infestation that may bias results. We included “sampling site” as a covariate because it is a likely source of variation but caution that the sampling is insufficient to draw any inferences about the sites themselves. Our response variable for *Bartonella* occurrence in individuals consisted of 0 s and 1 s (presence/absence). We restricted this analysis to the 10 species‐habitat‐site combinations that contained data from more than three individuals of the species, together comprising 196 individuals of five species captured across forest, grassland, and built‐up areas. Among habitat types, tea plantations are not represented in the analysis because for the 2 years of *Bartonella* data—2018 in Kadamane and 2021 in Kudremukh—we did not have any data from tea plantations. We found no small mammals in tea plantations in 2018 in Kadamane, and Kudremukh does not have tea plantations. We also examined habitat type as an additional covariate but dropped it because the model did not converge and because habitat type did not separately appear to show any correlation with prevalence when controlling for species identity.

### 2.6 Phylogenetic Analyses

We generated a concatenated alignment of partial *rpoB*, *ftsZ*, and *16SrRNA* sequences (1861 bp) with known *Bartonella* species sequences from the NCBI database and sequences reported in Ansil et al. 2021 (accession number MT785317‐ MT785361, MT787671‐ MT787733, & MT790782‐ MT790833) using MUSCLE in MEGA 7.0.26 (Kumar et al. 2016). Sequences generated from the ectoparasites were also included in this alignment. A subset of samples did not have all three gene sequences, and we retained them in the alignment. Prior to concatenation, individual phylogenies for *rpoB* and *ftsZ* were constructed (Figure S4), and their topologies were compared to assess the congruence and identify potential recombinant sequences. We detected five potentially recombinant sequences, which were subsequently removed from the concatenated alignment and all further analyses. We did not perform this analysis for 16S due to the missing reference sequences in the alignment. Missing data in one phylogeny can lead to incongruence in topology with other phylogenies.

Next, the concatenated alignment was codon‐optimized and tested for the appropriate nucleotide substitution models using PartitionFinder 2.1.1 (Lanfear et al. 2016). We observed four partitions in our dataset. We performed a Bayesian phylogenetic analysis using this alignment in BEAST 1.10.4 (Drummond and Rambaut 2007; Suchard et al. 2018), specifying the optimal nucleotide substitution model identified for each partition: (1) GTR + I + G + X, (2) HKY + I + G + X, (3) GTR + I + G + X, and (4) K80 + I + G. The phylogeny was run for 10^8^ Markov chain Monte Carlo (MCMC) cycles, and effective sample sizes (ESS) were assessed using Tracer 1.7.2 (Rambaut et al. 2018). The resulting trees were summarized into a consensus tree after a 25% burn‐in and were visualized and annotated using FigTree 1.4.4 (Rambaut 2014). The phylogenetic tree was rooted using *Brucella melitensis* (AY562181) as an outgroup. Internal nodes of the tree are marked and colored based on the posterior probability (PP).

### 2.7 Evolutionary Relationship Between *Bartonella* and Small Mammals

We created a separate *Bartonella* phylogeny for 15 unique genotypes (*rpoB*) recovered from multiple host species. We also incorporated *ftsZ* and *16S* sequences for a longer and more robust alignment (1861 bp). The optimal nucleotide substitution models for partitions one, two, and three (TRN + I + X, JC + I, and GTR + G + X) were specified, and a Bayesian phylogeny was constructed using BEAST 1.10.4 (Suchard et al. 2018). Similarly, we also constructed a phylogeny for six small mammal hosts using *Cytb* sequences (877 bp; GenBank accession number PV057291‐ PV057295 and HM217739), specifying three partitions and optimal substitution models (SYM + I, HKY + I + X, and HKY + G + X).

We applied a Procrustes Approach to Cophylogeny (PACo) using phylogenetic distance matrices to understand the dependence of *Bartonella* phylogeny (*rpoB, ftsZ, and 16S*) upon the host phylogeny and assess evolutionary codivergence and host shifts. We used the *paco* package (Hutchinson et al. 2017) and a Jackknife procedure in R to estimate the degree to which each small mammal‐Bartonella link (association) supported a hypothesis of phylogenetic congruence. Links were considered supportive of coevolution if their 95% confidence interval was below the mean of all squared Jackknife residuals (Balbuena et al. 2013; Becker et al. 2020).

Additionally, we performed a Bayesian phylogenetic ancestral trait (host) reconstruction analysis using the *Bartonella* sequences (15 sequences; 1861 bp). We used six identified host states as traits to estimate host probabilities for all ancestral nodes in BEAST 1.10.4 (Suchard et al. 2018). We employed the symmetric substitution model, which incorporates a continuous‐time Markov chain (CTMC) with reversible transition rates. The CTMC framework allows an equal likelihood for bidirectional transitions between host states. We also implemented the Bayesian Stochastic Search Variable Selection (BSSVS) procedure to limit the number of rates to those that sufficiently explained the phylogenetic diffusion process. We used default priors for host frequencies and transition rates (uniform and gamma distributions, respectively). The resultant phylogeny was evaluated and annotated as described before.

## 3 Results

### 3.1 Small Mammal Community

We recorded 11 small mammal species, of which nine were rodents and two were shrews (Table S1). The rodent species were *Rattus satarae* , *Rattus rattus* , *Mus cf. fernandoni*, *Mus cf. famulus*, *Mus cf. terricolor*, *Golunda ellioti* , *Vandeleuria nilagirica* , *Platacanthomys lasiurus* , and *Funambulus tristriatus* . The two shrew species were *Suncus niger* and *Crocidura horsfieldii* . The three *Mus* species were morphologically similar and could only be differentiated using their phylogenetic clustering (Figure S1). The average genetic distances (p distances) for *M. cf. fernandoni*, *M. cf. famulus*, and *M. cf. terricolor* from their respective parent species (e.g., *M. cf. fernandoni* vs. *M. fernandoni* ) were 0.05, 0.07, and 0.09, respectively. Due to limited sample size, we did not include rare species, such as *C. horsfieldii* , *G. ellioti* , and *V. nilagirica* , in any formal analysis. Additionally, some of these species were captured only during 2016 and 2017; hence, they were not sampled for *Bartonella* infection status.

**FIGURE 2:**
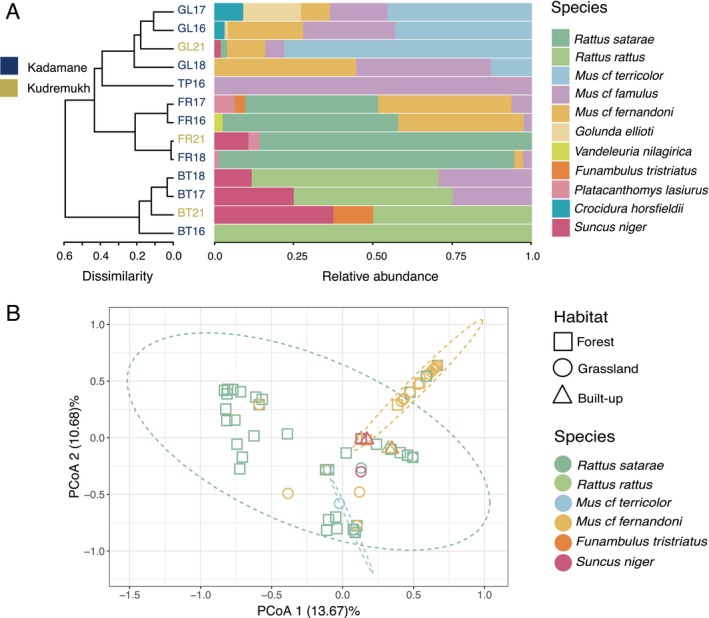
Community structure of small mammals and their ectoparasites. (A) Left: A dendrogram showing dissimilarity among small mammal communities inhabiting different habitats sampled and sampling years (2016, 2017, 2018, 2021). FR, Forest; GL, Grassland; TP, Tea plantation; and BT, Built‐up area. Right: Relative abundance of different small mammal species captured in different habitats. (B) Principal coordinate (PCoA) plot showing ectoparasite community structure across different small mammals and habitats. The ellipses represent the 95% confidence level of the respective sample group centroid. Some colors can appear similar but note that no individuals of *R. rattus* and *M. cf terricolor* were detected in forest.

Among the four different habitats sampled, forest and grassland had the highest species richness, each hosting seven small mammal species. Built‐up areas had four species detected, while tea plantations had only one species (*M. cf. famulus* captured once in 2016). Dissimilarity clustering revealed that the four forest small mammal communities (FR16, FR17, FR18, and FR21) formed a single cluster, varying by less than 20% across years and both sites (Figure 2A left). Communities in grassland and tea plantation (TP16) formed another cluster with 40% dissimilarity from the forest cluster. Grassland communities also varied by less than 20% across years (GL16, GL17, and GL18) and sites (GL21). Built‐up habitats were the most dissimilar (60%) to other land‐use types, although they retained two species ( *S. niger* and *M. cf. famulus*) detected in the others (Figure 2A right).

We were able to estimate the densities of four (most abundant of the 11) common small mammal species ( *R. satarae* , *M. cf. fernandoni*, *M. cf. famulus*, and *M. cf. terricolor*) in forest and grassland. Since the population was not “closed” between sampling occasions, we considered each sampling session (year) independently. These densities are reported in Supplementary results and Figure S2.

### 3.2 Ectoparasite Community

We found 10 ectoparasite morphotypes on the small mammals; two morphotypes of mites from the genus *Laelaps*, three of ticks, one each from the genera *Rhipicephalus*, *Ixodes*, and *Haemaphysalis*, and five of fleas from the genus *Xenopsylla* (Table S2). Since more than one morphotype (potentially species) was detected for the genera *Laelaps* (mites) and *Xenopsylla* (fleas), these morphotypes were named serially (e.g., *Xenopsylla sp 1, Xenopsylla sp 2* and so on). Six ectoparasite morphotypes (60%) were detected in multiple hosts (potentially generalists), and six of eight small mammals sampled (75%) were polyparasitic (infested with two or more ectoparasite morphotypes).

Our PCoA revealed substantial variation in the ectoparasite communities hosted by small mammals (Figure 2B, Figure S3). We did also observe similarities, however, in ectoparasite composition among individuals of common species (e.g., *R. satarae* and *M. cf. fernandoni*). *R. satarae* showed significant variation in the ectoparasite community along the PCoA axis 1 and 2 (13.67% and 10.68% explained, respectively), while *M. cf*. fernandoni predominantly displayed limited variation (similar ectoparasite communities dominated by *Laelaps* 1). Some individuals of *R. rattus* , *F. tristriatus* , *M. cf. fernandoni*, *M. cf. terricolor*, and *S. niger* had ectoparasite communities similar to those of some *R. satarae* . We also estimated the mean density of each ectoparasite species recovered from each small mammal species per habitat. These densities are reported in Supplementary results and Figure S3.

### 3.3 *Bartonella* Prevalence in Small Mammals

**FIGURE 3:**
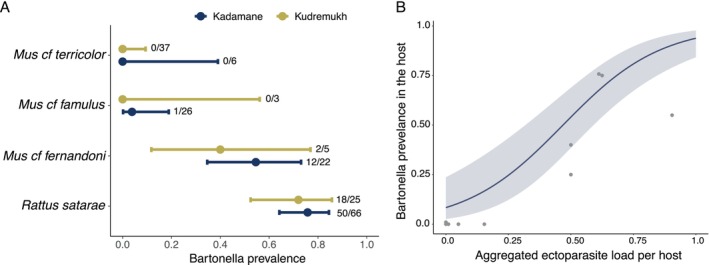
(A) *Bartonella* prevalence in the four most abundant small mammals. The number of positives and number of samples tested are indicated on the right side of the confidence interval. (B) *Bartonella* prevalence positively correlated with aggregated ectoparasite load within a small mammal species at each habitat‐site. The trend line indicates predicted *Bartonella* prevalence as a function of aggregated ectoparasite load. The shaded area represents the 95% confidence interval of the predicted prevalence estimate.

We detected *Bartonella* in five species of small mammals— *R. satarae* , *R. rattus* , *M. cf. fernandoni*, *M. cf. famulus*, and *S. niger* (Table 1). Prevalence varied considerably among these species (0%–75.8%), but was similar for each species at both sites (Figure 3A). *Bartonella* was detected most often in two species— *R. satarae* (75.8% (*n* = 66) in Kadamane and 72% (*n* = 25) in Kudremukh) and *M. cf. fernandoni* (54.5% (*n* = 22) in Kadamane and 40% (*n* = 5) in Kudremukh). The two remaining abundant species had low to zero prevalence—*M. cf. famulus* had 3.8% prevalence in Kadamane (*n* = 26) and 0% in Kudremukh (*n* = 3), while *M. cf. terricolor* had 0% prevalence at both sites (*n* = 6 and *n* = 37). Other species, such as *R. rattus* and *S. niger* , had low sample sizes; hence, we have low confidence in prevalence estimates.

**TABLE 1:** *Bartonella* prevalence in small mammal blood and ectoparasites.

	*Bartonella* prevalence—no. of positives/no. of samples tested (%)			
	Dried blood spot (DBS)		Pooled ectoparasites	
	Kadamane	Kudremukh	Kadamane	Kudremukh
*Rattus satarae*	50/66 (75.8)	18/25 (72)	24/71 (33.8)	6/24 (25)
*Rattus rattus*	0/10 (0)	1/4 (25)	—	0/3 (0)
*Mus cf. fernandoni*	12/22 (54.5)	2/5 (40)	9/29 (31)	0/6 (0)
*Mus cf. famulus*	1/26 (3.8)	0/3 (0)	0/1 (0)	—
*Mus cf. terricolor*	0/6 (0)	0/37 (0)	—	0/7 (0)
*Platacanthomys lasiurus*	—	0/1 (0)	—	0/1 (0)
*Funambulus tristriatus*	—	0/1 (0)	0/2 (0)	0/2 (0)
*Suncus niger*	0/2 (0)	2/4 (50)	0/1 (0)	1/2 (50)

*Note:* Prevalence reported for ectoparasites is not true prevalence since ectoparasites were pooled for screening (see Methods).

We found *Bartonella‐positive* ectoparasite pools typically on individual small mammals that were positive for *Bartonella*, except in five cases. Of these, one negative *R. satarae* had a negative *Rhipicephalus* sp. tick pool but a positive *Xenopsylla sp. 1* flea pool. Among the mite pools tested, *Laelaps sp. 1* collected from *M. cf. fernandoni* showed 25% positivity (*n* = 24), while none of the *Laelaps sp. 1* pools from *R. satarae* were positive (*n* = 3). Ticks were predominantly detected in *R. satarae* ; *Ixodes sp*. showed the highest positivity (58.8%, *n* = 34), followed by *Rhipicephalus sp*. (33.3%, *n* = 18) and *Haemaphysalis sp*. (4.5%, *n* = 22). Fleas were relatively rare, but *Xenopsylla sp. 1*, *Xenopsylla sp. 3*, and *Xenopsylla sp. 5* showed positivity in *R. satarae* (16.7%, *n* = 18), *M. cf. fernandoni* (33.3%, *n* = 3), and *S. niger* (100%, *n* = 1), respectively. Positivity rates of specific ectoparasite pools are provided in Supplementary results and Table S3.

### 3.4 Factors Affecting *Bartonella* Prevalence

*Bartonella* prevalence increased with aggregated ectoparasite load within a species at each habitat‐site combination (GLM, *p* < 0.05, Figure 3B). We did not find evidence that *Bartonella* prevalence was influenced by host densities and sampling locations (Table 2).

**TABLE 2:** Results of generalized linear model (GLM) examining correlates of *Bartonella* prevalence.

	Estimate	Std. error
Intercept	−1.633595	1.05266
Host density	−0.07574	0.06711
Site	−0.47880	0.72288
Aggregated ectoparasite load	5.10933*	0.96562

*Note:* Significant variables are marked with (*).

### 3.5 *Bartonella* Genotypes and Phylogenetic Relationship

**FIGURE 4:**
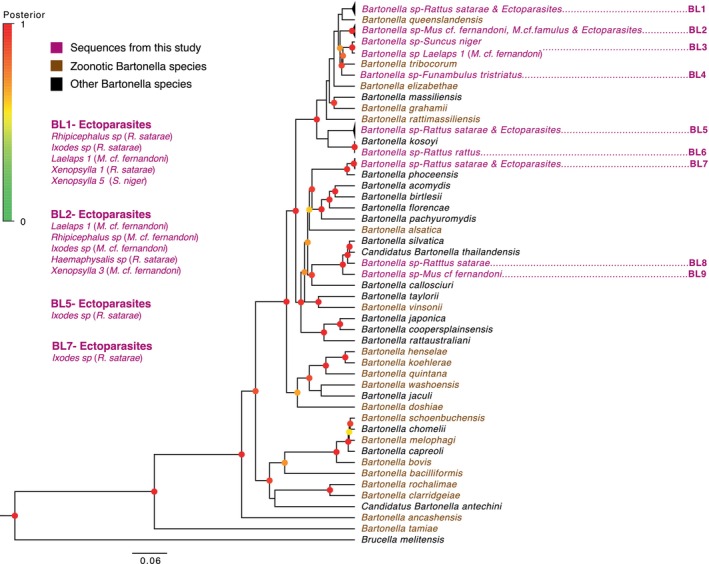
Bayesian phylogenetic inference of *Bartonella* based on 1861 bp of concatenated *rpoB, ftsZ*, and 16S sequences. Nodes are colored based on posterior probability (PP), and monophyletic clades are collapsed for easy visualization. PP < 0.2 is not displayed. Sequences from ectoparasites that are part of collapsed lineages, and their hosts are expanded on the left side. The gene phylogenies for *rpoB* and *ftsZ* (Figure S4), and the extended concatenated phylogeny (Figure S5) are provided as Supplementary Figures.

Based on *rpoB* sequences, we identified 15 distinct *Bartonella* genotypes, with more than one genotype linked to each of *R. satarae* and *M. cf. fernandoni* (Table S4). Phylogenetic analysis of concatenated *Bartonella* sequences (*rpoB, ftsZ, and 16S*; 1861 bp) revealed nine distinct lineages (BL1‐BL9) from small mammals and ectoparasites (Figure 4, Figure S5). We found that four lineages among them (BL1‐BL4) were phylogenetically related to known zoonotic *Bartonella* species. We observed four lineages in *R. satarae* (BL1, BL5, BL7, and BL8) and two lineages in *M. cf. fernandoni* (BL2 and BL9). BL1 included sequences from *R. satarae* and ectoparasites such as *Rhipicephalus sp*, *Ixodes sp*, *Laelaps sp 1, Xenopsylla sp 1*, and *Xenopsylla sp 5*. While *Rhipicephalus sp, Ixodes sp, and Xenopsylla sp 1* were collected from *R. satarae* , *Laelaps sp 1*, and *Xenopsylla sp 5* were collected from *M. cf. fernandoni* and *S. niger* , respectively, indicating a nonspecific association of *Bartonella* and ectoparasites. This lineage displayed phylogenetic relatedness to *B. queenslandensis* , a known zoonotic species. BL2 consisted of sequences from *M. cf. fernandoni*, *M. cf. famulus*, and ectoparasites (*Laelaps sp 1, Rhipicephalus sp, Ixodes sp, Haemaphysalis sp*, and *Xenopsylla sp 3*). In this lineage, the *Haemaphysalis sp* was recovered from *R. satarae* and not from any of the *Mus* species (nonspecific ectoparasite‐*Bartonella* association as observed previously). BL3 consisted of two sequences, one from *S. niger* and the other from *Laelaps sp 1* collected from *M. cf. fernandoni*. These two lineages (BL2 and BL3) showed phylogenetic affinity with *B. tribocorum* , another known zoonotic species. The single sequence from *F. tristriatus* (reported in Ansil et al. 2021; accession number—MT787671) formed a basal lineage (BL4) to BL3 while maintaining a phylogenetic affinity with *B. tribocorum* . We included this sequence in our analysis since it was collected during our study in Kadamane.

The remaining five lineages (BL5‐BL9) were associated with nonzoonotic *Bartonella* species. Notably, BL5 and BL6 emerged as sister lineages; BL5 comprised sequences recovered from *R. satarae* and *Ixodes* ticks collected from them, while BL6 included a single sequence from *R. rattus* along with a recently identified *B. kosoyi* (Gutiérrez et al. 2020). Similar to BL5, BL7 comprised sequences from *R. satarae* (third lineage from *R. satarae* ) and *Ixodes sp*. recovered from them. This lineage was related to *B. phoceensis*. The BL8 and BL9 lineages were distinct, with BL9 forming a basal lineage to BL8. These lineages included a single sequence from *R. satarae* (the fourth lineage from *R. satarae* ) and *M. cf. fernandoni* (the second lineage from *M. cf. fernandoni*), respectively. Both of these lineages were phylogenetically related to *B. thailandensis* and *B. silvatica* .

### 3.6 *Bartonella*‐Small Mammal Evolutionary Association

**FIGURE 5:**
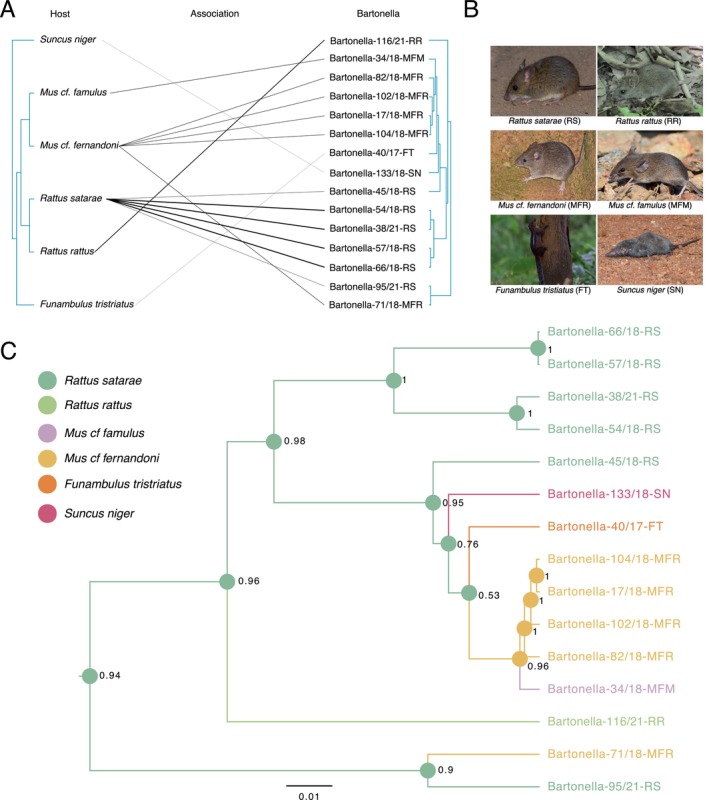
(A) Cophylogenetic association of small mammals and *Bartonella* genotypes. Lines are shaded by the inverse of the squared residuals from *PACo*. Links with smaller residuals show darker lines, indicative of coevolution. (B) Images of different hosts detected with *Bartonella* in the community. (C) Bayesian phylogenetic inference showing ancestral host state of different *Bartonella* genotypes. Color of the common ancestor nodes represents specific hosts, and the number next to the nodes indicates the probability of the corresponding host state.

Our coevolutionary analysis (PACo) did not support congruence between small mammal and *Bartonella* phylogenies (m^2^_XY_ = 30.79, *p* = 0.25, *n* = 1000; Figure 5A), suggesting *Bartonella* evolution is independent of host speciation. However, four genotypes (Bartonella‐38/21‐RS, Bartonella‐57/18‐RS, Bartonella‐66/18‐RS and Bartonella‐54/18‐RS) and their small mammal association links supported coevolution (Figure 5A). The remaining 11 unique associations (including Suncus niger−Bartonella‐133/18‐SN and *Funambulus tristriatus* − Bartonella‐40/17‐FT) did not support coevolution and thus indicate probable host shifts (95% confidence interval greater than mean squared Jackknife residuals, Figure S6). We further validated these host shift signals with a Bayesian phylogenetic ancestral trait (host) reconstruction (Figure 5C). The results indicated *R. satarae* as the ancestral host for three unique genotypes not associated with *R. satarae* (Bartonella‐133/18‐SN, Bartonella‐116/21‐RR, Bartonella‐71/18‐MFR; host probability > 0.7), once again suggesting historic host shifts. Similarly, we found *M. cf. fernandoni* as an ancestral host for Bartonella‐34/18‐MFM (host probability = 0.96) suggesting a historic host shift between *M. cf. fernandoni* and *M. cf. famulus*. However, we found no evidence for contemporary *Bartonella* sharing among small mammal hosts in the community despite nonspecific *Bartonella* and ectoparasite associations (Figure 4, Figure S5).

## 4 Discussion

### 4.1 *Bartonella* Associations Are Species‐Specific with Small Mammals but Nonspecific with Ectoparasites

Characterizing *Bartonella* diversity and its association with small mammals and ectoparasites is crucial from a public health perspective, especially because many *Bartonella* species are zoonotic and can sometimes cause life‐threatening endocarditis in humans (Saisongkorh et al. 2009). For instance, endemic *Bartonella* variants such as *B. bacilliformis* , responsible for Carrion's disease, can be fatal and cause severe mortality (Garcia‐Quintanilla et al. 2019). Among the nine lineages we identified in this study, four share a close evolutionary relationship with known zoonotic species such as *B. queenslandensis* , *B. tribocorum* , and *B. elizabethae* . Previous studies have documented the widespread presence and global circulation of these *Bartonella* species, primarily facilitated by small mammals and domestic animals (Frank et al. 2018). However, it is important to note that phylogenetic similarity alone does not confirm their ability to cause disease in humans. Additional validation through molecular and in vitro experiments is warranted to establish their pathogenicity in humans and other mammalian hosts (Che et al. 2014; Saenz and Dehio 2005).

We observed contrasting patterns of association with *Bartonella* for small mammals and ectoparasites. As expected based on previous studies (Ansil et al. 2021; Lei and Olival 2014; Withenshaw et al. 2016), *Bartonella* had species‐specific associations with the small mammal hosts in our study region. However, *Bartonella* had nonspecific associations with ectoparasite vectors (e.g., *Xenopsylla sp* 5 collected from *S. niger* carried *Bartonella* associated with *R. satarae* ), leading to a higher overall diversity of *Bartonella* genotypes in each ectoparasite morphotype compared to each small mammal species. These interesting patterns are not unprecedented, as they have been documented elsewhere (Silaghi et al. 2016; Withenshaw et al. 2016), but the mechanisms that drive this nonspecific association and persistence of *Bartonella* in ectoparasites remain unclear. We therefore advise caution in interpreting these findings, as the ectoparasites may have acquired *Bartonella* from previous blood meals on a different host (transstadial transmission) or through vertical transovarial transmission (Stich et al. 2008). Nevertheless, nonspecific associations between *Bartonella* and ectoparasites have significance for potential cross‐species transmission and host shifts, given vector competency and favorable ecological conditions (Borremans et al. 2019).

### 4.2 *Bartonella* Prevalence and Transmission May Be Mediated by Ectoparasites

Our results suggest that nonspecific ectoparasites may be a key element in mediating transmission pathways and *Bartonella* prevalence in this host–ectoparasite–pathogen system. We had expected host density to be a primary driver of *Bartonella* prevalence (Rodríguez‐Pastor et al. 2019), but instead found a strong correlation between prevalence and ectoparasitism (aggregated ectoparasite load). This correlation holds ecological and epidemiological significance. Because *Bartonella* is a vector‐borne pathogen known for its transmission through blood‐feeding arthropods (Moreno Salas et al. 2019), elevated densities of infected ectoparasites on a specific host can increase transmission probabilities and impact prevalence and the risk of infection (Kantsø et al. 2010). Similarly, high ectoparasite loads in the environment can increase chances of *Bartonella* transmission among hosts when there is a high degree of polyparasitism (a single host associated with multiple vectors) and host sharing (a single vector associated with multiple hosts). We found widespread evidence of both polyparasitism and host sharing in our study system. Polyparasitism and host sharing can together create a transmission pathway (via ectoparasite vectors) across habitats, if small mammals were to move across habitat boundaries.

Many small mammal species from the study were polyparasitic. Of particular significance is the polyparasitism shown by *R. satarae* and *M. cf. fernandoni*. Both are relatively large‐bodied and abundant species that have high ectoparasite densities and high *Bartonella* prevalence. Other studies have found a similar positive correlation between body size and ectoparasitism (Esser et al. 2016). When abundant polyparasitic species share space (habitat) with other small mammals, they can facilitate the acquisition of novel ectoparasites and drive overall ectoparasite richness in the entire small mammal community (Bordes et al. 2015; Dáttilo et al. 2020). Moreover, several ectoparasites (e.g., *Rhipicephalus sp*, *Xenopsylla sp 1*) were found across a range of small mammal hosts, presumably due to their ability to infect multiple locally available hosts (McCoy et al. 2013), supporting the recent understanding that host sharing among vectors is more common than previously believed (Goldberg et al. 2020). Such nonspecific host–ectoparasite associations can facilitate the transmission of several pathogens, making their ecology a crucial part of vector‐borne pathogen dynamics (Sotomayor‐Bonilla et al. 2019).

While these insights are valuable, it is worth noting that some variables previously shown to influence pathogen dynamics, like seasonality (Rodríguez‐Pastor et al. 2019) and climatic variables such as temperature, precipitation, and humidity (Zhang et al. 2024), were not included in this study. We urge follow‐up studies to incorporate these variables at local scales to elucidate further nuance into the drivers of pathogen prevalence and transmission. Additionally, low sample sizes for some hosts and ectoparasites limited our capacity to comprehensively assess polyparasitism and host sharing across the entire small mammal–ectoparasite system. We especially lacked substantive data for the less common species, but adequate representation of rare species is an inherent challenge in studying natural communities (Chiarucci et al. 2011). More targeted sampling efforts focused specifically on rare species are necessary to enhance sample sizes and more accurately characterize ectoparasite–host associations. Moreover, given the complex life cycles of ectoparasites (e.g., larval and nymph stages), characterizing them at the species level using molecular methods such as DNA barcoding would provide a more accurate understanding of host sharing, and therefore, transmission pathways.

### 4.3 Some Small Mammals May Serve as Potential *Bartonella* Carriers Across Habitats

Do small mammal host species move between habitats, thereby facilitating potential transmission of their specialized *Bartonella* variants to novel hosts via ectoparasites? We observed strong species–habitat associations in the focal small mammal community, characterized by exclusive occurrences and high densities, mirroring several ecological communities (Hill et al. 2008; Stephens and Anderson 2014). While species like *R. satarae* (a major *Bartonella* host with the highest prevalence) showed high abundance and nearly exclusive occurrence in forested habitats, *R. rattus* , a known synanthropic species, was only detected in built‐up areas, reaffirming its commensal nature. Similarly, *M. cf. terricolor* was exclusively recorded in grasslands in both sites, albeit with higher density in Kudremukh. Other *Mus* species, *M. cf. fernandoni* and *M. cf. famulus*, were also abundant in grasslands, indicating a generalizable association of *Mus* species with grassland ecosystems. Interestingly, all three *Mus* species in Kadamane showed variation in annual density, indicating annual population oscillations (Lindström et al. 2001; Myers 2018). We could not fully capture this pattern due to the relatively short duration of this study, but we urge future studies to investigate long‐term trends in host densities as they are linked to zoonotic hazard and spillover (Gibb, Franklinos, et al. 2020; Plowright et al. 2017).

We found evidence, however, that species do cross habitat boundaries in this landscape. Among the 11 species recorded during the study, five (45%) were recorded in more than one of the following habitats—forest, grassland, and built‐up habitat. Of these, *M. cf. famulus* and *S. niger* were recorded in all habitats, indicating that they may be habitat generalists. The occurrence of a species in multiple habitats, albeit at varying abundances, holds significance, particularly in the context of interspecies transmission pathways both within and among different habitat types (Boyard et al. 2008). *M. cf. fernandoni* was found to occur at low densities in forest in addition to grassland, bringing it in contact with *R. satarae* , potentially paving the way for cross‐species and crosshabitat transmission via shared ectoparasites. Significantly, species with low *Bartonella* prevalence like *M. cf. famulus* can also be incidental hosts (as may be the case with the single positive) or just carriers of positive ectoparasites, connecting potential reservoir hosts like *R. satarae* and *M. cf. fernandoni*, with the synanthropic *R. rattus* (another seemingly incidental host with a single positive) and humans. Risks of *Bartonella* transmission to humans and domestic animals may be mediated especially by generalist fleas (Moreno Salas et al. 2019). We understand from this study that *R. rattus* hosts multiple generalist fleas that can, in theory, move between wild and synanthropic small mammals (including *R. rattus* ), thereby bringing *Bartonella* infection to human habitation.

Significantly, we also find that small mammals in this part of the Western Ghats are in urgent need of taxonomic revision. Previous studies from the landscape morphologically identified *M. cf. fernandoni* as *M. musculus* and *M. cf. famulus* and *M. cf*. *terricolor* as *M. booduga* (Ansil et al. 2021). Phylogenetic analysis and genetic distances suggest that these are distinct lineages related to *M. fernandoni* , *M. famulus* , and *M. terricolor* , respectively. We urge researchers to sample rodents in this landscape and resolve their taxonomy on priority because pathogen and habitat associations are often linked with species identity.

### 4.4 Evidence of Historic Host Shifts Suggests Future Spillover Risk

We found, through cophylogenetic analysis, that the evolution of *Bartonella* does not align with that of its small mammal hosts (only four lineages showed support for coevolution), indicating multiple host shift events, even among distantly related species. Previous studies on other blood‐borne, vector‐transmitted pathogens have also documented instances of such host shifts among distantly related host species (Becker et al. 2020). Phylogenetic ancestral trait reconstruction provided an additional line of evidence that cross‐species transmission may have already occurred in the past within this small mammal community. Such host shifts may perhaps be expected for *Bartonella*, because it is a vector‐borne pathogen that can be carried by generalist ectoparasites that parasitize multiple small mammal species (Billeter et al. 2008).

We observed *R. satarae* as the ancestral host for several *Bartonella* lineages with a high probability, indicating host shifts or historic spillover events involving this species. This was evident in many genotypes, including the ones recovered from *R. satarae* (Bartonella‐95/21‐RS) and *M. cf. fernandoni* (Bartonella‐71/18‐MFR; Figure 5C). Interestingly, these two species stood out as the primary *Bartonella* hosts in our dataset, displaying high prevalence, high genotype diversity, and co‐occurrence in forested habitats. Such pathogen movement among host species could provide an opportunity to evolve new virulent variants through recombination (Borremans et al. 2019), similar to those observed in other pathogen systems (Wang and Eaton 2007). Although we did not find instances of contemporary *Bartonella* sharing, the historic host shifts, potentially through ectoparasites, emphasized the likelihood of similar spillover events in the future. Moreover, the movement of generalist hosts across multiple habitats carrying generalist ectoparasites with nonspecific *Bartonella* associations is likely to further drive pathogen spillover in the landscape (Borremans et al. 2019).

## 5 Conclusion

Our study has uncovered significant insights about the intricate relationships among small mammal hosts, their ectoparasites, and bacterial agents within a tropical mixed‐use landscape. While our model system and investigation were specific to *Bartonella*, the results and inferences extend to a broader spectrum of vector‐borne bacterial pathogens in multihost communities. Our research highlights the crucial role of ectoparasitism in driving *Bartonella* prevalence within small mammal communities. Because ectoparasite associations with *Bartonella* and small mammals are nonspecific compared to specialized small mammal–*Bartonella* associations, the movement of generalist hosts across multiple habitats can facilitate pathogen spillover. When habitats are modified, and habitat boundaries and edges are extensive, the risk of small mammals transcending boundaries and causing spillover events may be enhanced. We further demonstrate that such spillover events that occurred in the past may have shaped the current *Bartonella* distribution in the community. Collectively, our findings underscore the need for comprehensive ecological studies to advance our understanding of the transmission dynamics of vector‐borne bacterial pathogens within multihost communities and their zoonotic implications, particularly in rapidly changing landscapes.

## Author Contributions

**B. R. Ansil:** conceptualization (equal), data curation (lead), formal analysis (lead), investigation (lead), methodology (lead), project administration (lead), visualization (lead), writing – original draft (lead), writing – review and editing (lead). **Ashwin Viswanathan:** data curation (equal), formal analysis (supporting), methodology (supporting), visualization (supporting), writing – review and editing (equal). **Vivek Ramachandran:** investigation (supporting), methodology (supporting), writing – review and editing (equal). **H. M. Yeshwanth:** investigation (supporting), methodology (supporting), visualization (supporting), writing – review and editing (equal). **Avirup Sanyal:** investigation (supporting), methodology (supporting), writing – review and editing (equal). **Uma Ramakrishnan:** conceptualization (equal), funding acquisition (lead), project administration (lead), supervision (lead), writing – review and editing (equal).

## Ethics Statement

This study and associated protocols were approved by the National Centre for Biological Science Institutional Animal Ethics Committee (NCBS‐IAEC‐2016/10‐[M], NCBS‐IAE‐2020/02[N]) and the Institutional Biosafety Committee (TFR:NCBS:23_IBSC/2017).

## Conflicts of Interest

The authors declare no conflicts of interest.

## Supporting information


Data S1.

Data S2.

Data S3.

## Data Availability

The manuscript and Supporting Information S1and S2 present small mammal capture data and *Bartonella* prevalence data from small mammals and ectoparasites. *Bartonella* sequence data generated during this study are deposited in GenBank under the accession numbers OR574780 –OR574826, PP800974 –PP800985, PP799247 –PP799254.
