# Supplementary material for: Host–Pathogen–Vector Continuum in a Changing Landscape: Potential Transmission Pathways for Bartonella in a Small Mammal Community
Source: Ecol Evol. 2025 Apr 2;15(4):e71085. doi: 10.1002/ece3.71085 (PMC11962204; doi:10.1002/ece3.71085)
Supplement: Supplementary file 2 — Data S2. [file ECE3-15-e71085-s001.docx]

Table S1: Small mammals captured in different land-use types across different years. Year 2016, 2017, and 2018 represent samples from Kadamane and 2021 represents samples from Kudremukh National Park.

| **Species** | **Forest** | | | | **Grassland** | | | | **Tea plantation** | | | **Built-up** | | | | **Total** |
| --- | --- | --- | --- | --- | --- | --- | --- | --- | --- | --- | --- | --- | --- | --- | --- | --- |
|  | **2016** | **2017** | **2018** | **2021** | **2016** | **2017** | **2018** | **2021** | **2016** | **2017** | **2018** | **2016** | **2017** | **2018** | **2021** |  |
| *Rattus satarae* | 21 | 13 | 67 | 24 | - | - | - | 1 | - | - | - | - | - | - | - | **126** |
| *Rattus rattus* | - | - | - | - | - | - | - | - | - | - | - | 4 | 2 | 10 | 4 | **20** |
| *Mus cf. fernandoni* | 15 | 13 | 2 | - | 22 | 1 | 21 | 6 | - | - | - | - | - | - | - | **80** |
| *Mus cf. famulus* | 1 | 2 | 2 | - | 27 | 2 | 20 | 3 | 3 | - | - | - | 1 | 5 | - | **66** |
| *Mus cf. terricolor* | - | - | - | - | 40 | 5 | 6 | 39 | - | - | - | - | - | - | - | **90** |
| *Golunda ellioti* | - | - | - | - | 1 | 2 | - | - | - | - | - | - | - | - | - | **3** |
| *Vandeleuria nilagirica* | 1 | - | - | - | - | - | - | - | - | - | - | - | - | - | - | **1** |
| *Platacanthomys lasiurus* | - | 2 | 1 | 1 | - | - | - | - | - | - | - | - | - | - | - | **4** |
| *Funambulus tristriatus* | - | 1 | - | - | - | - | - | - | - | - | - | - | - | - | 1 | **2** |
| *Suncus niger* | - | - | - | 3 | - | - | - | 1 | - | - | - | - | 1 | 2 | 3 | **10** |
| *Crocidura horsfieldii* | - | - | - | - | 3 | 1 | - | - | - | - | - | - | - | - | - | **4** |
| **Total** | **38** | **31** | **72** | **28** | **93** | **11** | **47** | **50** | **3** | **-** | **-** | **4** | **4** | **17** | **8** | **406** |

Table S2: Ectoparasite occurrence in small mammal species. The number indicates number of occasions a specific morphotype (species) was observed in a host species. The percentage indicate ectoparasite load on a specific species (equivalent to prevalence).

|  | **Ectoparasites occurrence in small mammals (%)** | | | | | | | | |
| --- | --- | --- | --- | --- | --- | --- | --- | --- | --- |
|  | *Rattus satarae*  *n=92* | *Rattus rattus*  *n=14* | *Mus cf. fernandoni*  *n=29* | *Mus cf. famulus*  *n=30* | *Mus cf. terricolor*  *n=45* | *Platacanthomys. lasiurus*  *n=2* | *Funambulus tristriatus*  *n=1* | *Suncus niger*  *n=9* | Total  *n=222* |
| *Laelaps sp 1* | 3 (3.3) | - | 24 (82.8) | - | - | - | - | - | 27 (12.2) |
| *Laelaps sp 2* | - | - | - | - | - | 1 (50) | - | - | 1 (0.5) |
| *Rhipicephalus sp* | 18 (19.6) | - | 4 (13.8) | - | 5 (11.1) | - | - | 1 (11.1) | 28 (12.6) |
| *Ixodes sp* | 34 (37) | - | 2 (6.9) | - | - | - | - | - | 36 (16.2) |
| *Haemaphysalis sp* | 22 (23.9) | - | 2 (6.9) | - | 2 (4.4) | - | - | - | 26 (11.7) |
| *Xenopsylla sp 1* | 18 (19.6) | 1 (7.1) | - | - | - | - | 1 (100) | - | 20 (9) |
| *Xenopsylla sp 2* | - | 1 (7.1) | - | - | - | - | - | - | 1 (0.5) |
| *Xenopsylla sp 3* | - | - | 3 (10.3) | 1 (3.3) | - | - | 1 (100) | 1 (11.1) | 6 (2.7) |
| *Xenopsylla sp 4* | - | 1 (7.1) | - | - | - | - | - | - | 1 (0.5) |
| *Xenopsylla sp 5* | - | - | - | - | - | - | - | 1 (11.1) | 1 (0.5) |

Table S3: *Bartonella* positivity in various ectoparasites (pooled) recovered from small mammal species. Since ectoparasites collected were pooled for screening, positivity reported here is not true prevalence in the ectoparasite community.

|  | ***Bartonella* positivity in pooled ectoparasites; number of positives/number of pools tested (%)** | | | | | | | | |
| --- | --- | --- | --- | --- | --- | --- | --- | --- | --- |
|  | *Rattus satarae* | *Rattus rattus* | *Mus cf. fernandoni* | *Mus cf. famulus* | *Mus cf. terricolor* | *Platacanthomys lasiurus* | *Funambulus tristriatus* | *Suncus niger* | Total |
| *Laelaps sp 1* | 0/3 (0) | - | 6/24 (25) | - | - | - | - | - | 6/27 (22.2) |
| *Laelaps sp 2* | - | - | - | - | - | 0/1 (0) | - | - | 0/1 (0) |
| *Rhipicephalus sp* | 6/18 (33.3) | - | 1/4 (25) | - | 0/5 (0) | - | - | 0/1 (0) | 7/28 (25) |
| *Ixodes sp* | 20/34 (58.8) | - | 1/2 (50) | - | - | - | - | - | 21/36 (58.3) |
| *Haemaphysalis sp* | 1/22 (4.5) | - | 0/2 (0) | - | 0/2 (0) | - | - | - | 1/26 (3.8) |
| *Xenopsylla sp 1* | 3/18 (16.7) | 0/1 (0) | - | - | - | - | 0/1 (0) | - | 3/20 (15) |
| *Xenopsylla sp 2* | - | 0/1 (0) | - | - | - | - | - | - | 0/1 (0) |
| *Xenopsylla sp 3* | - | - | 1/3 (33.3) | 0/1 (0) | - | - | 0/1 (0) | 0/1 (0) | 1/6 (16.7) |
| *Xenopsylla sp 4* | - | 0/1 (0) | - | - | - | - | - | - | 0/1 (0) |
| *Xenopsylla sp 5* | - | - | - | - | - | - | - | 1/1 (100) | 1/1 (100) |

Table S4: Unique Bartonella genotypes (*rpoB*) identified from six species of small mammals in the community. These genotypes were used after incorporating more data from *ftsZ* and *16S* loci for co-phylogenetic analysis and ancestral trait reconstruction.

| **Small mammal species** | **Number of genotypes** | **Name of the genotypes** |
| --- | --- | --- |
| *Rattus satarae* | 6 | Bartonella-45/18-RS  Bartonella-54/18-RS  Bartonella-57/18-RS  Bartonella-66/18-RS  Bartonella-38/21-RS  Bartonella-95/21-RS |
| *Rattus rattus* | 1 | Bartonella-116/21-RR |
| *Mus cf. fernandoni* | 5 | Bartonella-17/18-MFR  Bartonella-71/18-MFR  Bartonella-82/18-MFR  Bartonella-102/18-MFR  Bartonella-104/18-MFR |
| *Mus cf. famulus* | 1 | Bartonella-34/18-MFM |
| *Funambulus tristriatus* | 1 | Bartonella-40/17-FT |
| *Suncus niger* | 1 | Bartonella-133/18-SN |
| **Total** | **15** |  |
