# Supplementary material for: Host–Pathogen–Vector Continuum in a Changing Landscape: Potential Transmission Pathways for Bartonella in a Small Mammal Community
Source: Ecol Evol. 2025 Apr 2;15(4):e71085. doi: 10.1002/ece3.71085 (PMC11962204; doi:10.1002/ece3.71085)
Supplement: Supplementary file 3 — Data S3. [file ECE3-15-e71085-s003.docx]

Figure S1: Phylogenetic­ inference of *Mus* species. This phylogenetic tree based on 870 bp of *cytochrome B* gene was used to identify cryptic Mus species from the study area (Western Ghats). Representative sequences are available under GenBank accession number PV057294- PV057299.

Figure S2: Estimated density of the four most common small mammals in the community. Points without error bars indicate number of individuals detected per hectare (due to low sample size or lack of recaptures). In 2018, individuals were sacrificed for organ samples, hence no recaptures for density estimates.

**
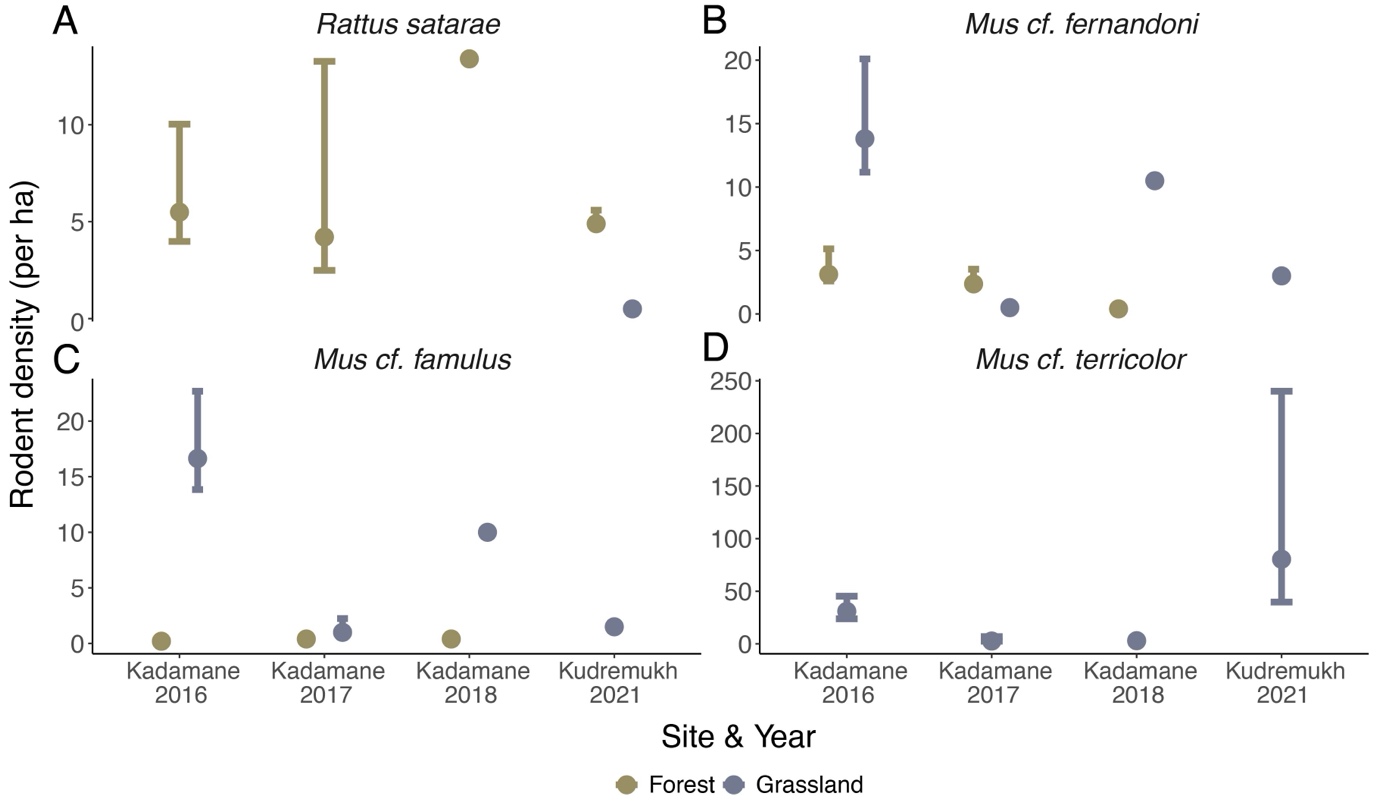
**

Figure S3: Estimated densities of various ectoparasites collected from small mammals. A, B and C show densities of various species of mites, ticks and fleas, respectively. The numbers next to the points in B and C are unusual ectoparasites counts observed manually labelled to preserve Y-axis distribution. D shows microscopic images (not to scale) of different ectoparasites collected from small mammals in the study area.


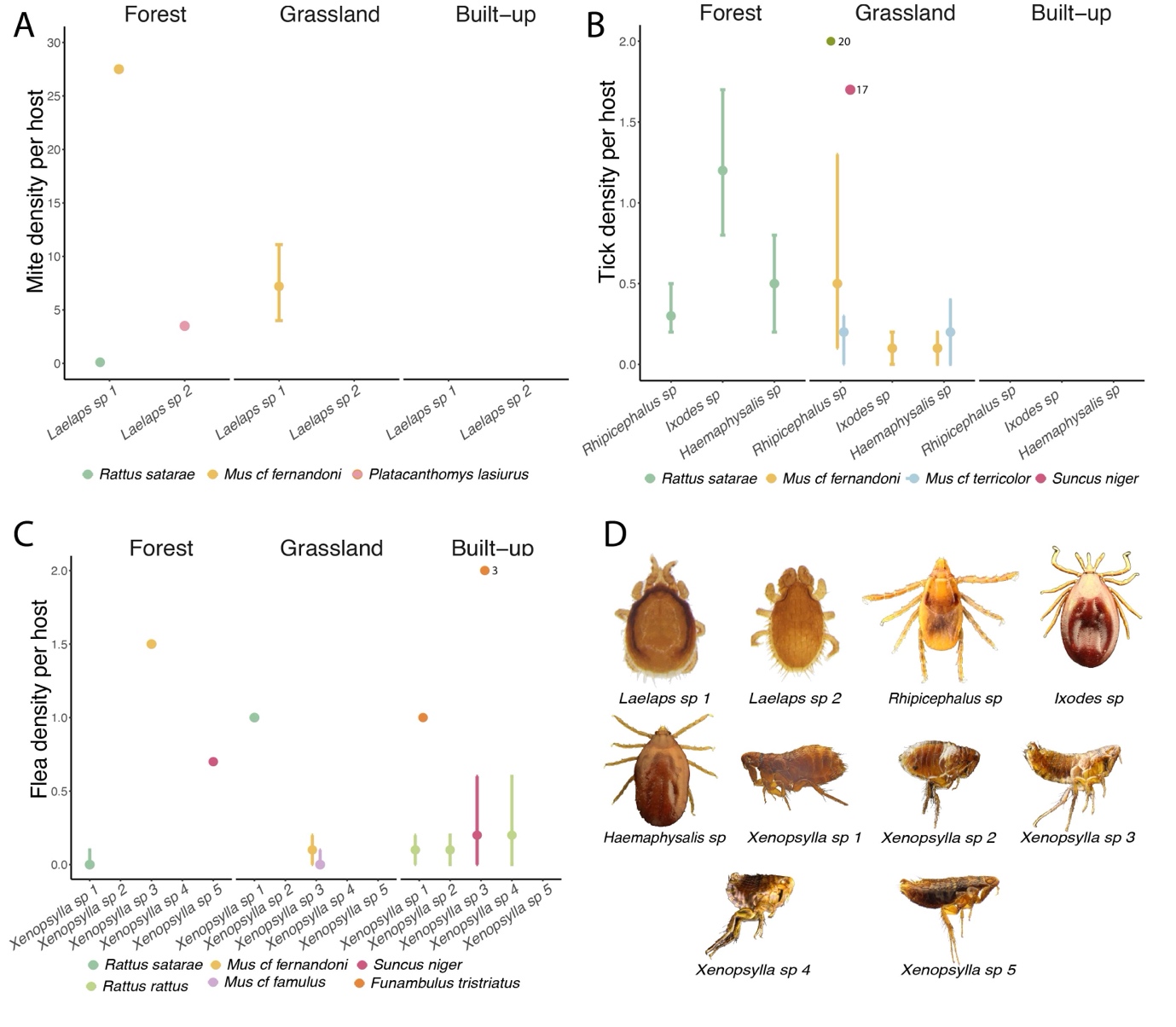


Figure S4: Bayesian phylogenetic inference of *Bartonella* based on (A) *rpoB* and (B) *ftsZ* sequences. Nodes are color-coded according to posterior probability (PP). Low PPs for internal nodes within lineages (i.e., highly similar sequences) are omitted for clarity. Five potential recombinant sequences, which were excluded from the concatenated sequence analyses, are marked with an asterisk (*) in the *ftsZ* phylogeny.

Figure S5: Bayesian phylogenetic inference of *Bartonella* based on 1861 bp of concatenated *rpoB, ftsZ,*and 16S sequences. Nodes are color-coded according to posterior probability (PP). Low PPs for internal nodes within lineages (i.e., highly similar sequences) are omitted for clarity.

Figure S6. Jackknife squared residuals (bars) and upper 95% confidence intervals (error bars) associated with each small mammal-Bartonella link. If the upper 95% confidence interval falls below the mean squared residuals (shown in the dashed line), the links are considered supportive of coevolution.
